# Factors associated with long turnaround time for early infant diagnosis of HIV in Myanmar

**DOI:** 10.1080/16549716.2017.1395657

**Published:** 2017-11-08

**Authors:** Soe Thiha, Hemant Deepak Shewade, Sairu Philip, Thet Ko Aung, Nang Thu Thu Kyaw, Myo Minn Oo, Khine Wut Yee Kyaw, May Wint War, Htun Nyunt Oo

**Affiliations:** ^a^ HIV Unit, International Union Against Tuberculosis and Lung Disease (The Union), Mandalay, Myanmar; ^b^ Department of Operational Research, International Union Against Tuberculosis and Lung Disease (The Union), New Delhi, India; ^c^ Department of Community Medicine, Government T.D. Medical College, Alappuzha, India; ^d^ Department of Operational Research, International Union Against Tuberculosis and Lung Disease (The Union), Mandalay, Myanmar; ^e^ Public Health Laboratory, Department of Medical Services, Ministry of Health and Sports, Mandalay, Myanmar; ^f^ National AIDS Program, Department of Public Health, Ministry of Health and Sports, Nay Pyi Taw, Myanmar

**Keywords:** early diagnosis/utilization, turnaround time, HIV-exposed babies, operational research, SORT IT

## Abstract

**Background**: A previous review of early infant diagnosis (EID) using polymerase chain reaction technology (PCR) under integrated HIV care (IHC) program in Myanmar revealed a low uptake of timely (within 6 to 8 weeks of babies’ age) EID and a long turnaround time (TAT) of receiving results.

**Objective**: This study aimed to determine the proportion and factors associated with the composite outcome of a long TAT (≥7 weeks; from sample collection to receipt of result by mother) or nonreceipt of result among HIV-exposed babies whose blood samples were collected for PCR at <9 months of age under the IHC program, Myanmar (2013–15).

**Methods**: Cohort study involving record review of routinely collected data. A predictive Poisson regression model with robust variance estimates was fitted for risk factors of long TAT or nonreceipt of result.

**Results**: Blood samples of 1 000 babies were collected; among them, long TAT or nonreceipt of results was seen in 690 (69%), and this was more than 50% across all subgroups. Babies with a mother’s CD4 count of 100–350 cells/mm^3^ at enrollment [adjusted RR (0.95 confidence intervals, CI): 0.8 (0.7, 0.9)] had a 20% lower risk of long TAT or nonreceipt of results when compared with ≥350 cells/mm^3^. Distance between ART center and PCR facility ≥105 km [adjusted RR (0.95 CI): 1.2 (1.1, 1.4)], when compared with <105 km, was associated with 20% higher risk of long TAT or nonreceipt of results.

**Conclusions**: The proportion of babies with long TAT or nonreceipt of result by the mother was high. Point-of-care testing for EID may reduce TAT/nonreceipt of results by the mother. Health system, laboratory, and logistic factors such as sample transportation, laboratory procedures, and result dispatching associated with long TAT should be further explored.

## Background

Of 1.8 million children (aged < 15 years) living with human immunodeficiency virus (HIV) worldwide, 111 000 children died from AIDS-related illnesses in 2015 [1]. More than 90% of them acquired HIV through mother-to-child transmission [2]. Early infant diagnosis (EID) is one of the crucial steps in the prevention of mother to child transmission programs (PMTCT). EID is the first critical step for babies born to mothers living with HIV (HIV-exposed babies) to get diagnosis and access to treatment as early as possible. Without early treatment, one-third of the babies living with HIV die within one year [3]. Hence, World Health Organization (WHO) recommended EID in HIV-exposed babies at 6–8 weeks age using a polymerase chain reaction (PCR) technology [4,5].

However, the proportion of HIV-exposed babies receiving timely EID (within 6–8 weeks) in many low- and middle-income countries was about 42%, and there were factors associated with low uptake of EID [6–8]. This is not an exception in Myanmar, which is one of the low- and middle-income countries. Of 9 482 children living with HIV in Myanmar at the end of 2015, 2 169 HIV-exposed babies received Nevirapine prophylaxis under PMTCT program, and only 801 of them received timely EID, suggesting a high loss to follow up and/or delay in accessing EID [9].

The International Union Against Tuberculosis and Lung Disease (The Union) has been implementing an integrated HIV care (IHC) program in Myanmar in collaboration with the national AIDS program (NAP). In March 2012, The Union, in collaboration with Fondation Merieux, provided logistic and technical support to set up a PCR facility (HIV DNA and RNA viral load) at the Public Health Laboratory (PHL) in Mandalay, the third largest city in Myanmar [10]. Since 2012, the IHC program provided ART and EID through its ART centers according to WHO recommendations [4].

In our previous study on uptake of EID under the IHC program (2013–2015), there was a low uptake of timely EID (47%) and a long median turnaround time (TAT) from sample collection to result receipt by mother in the program. Timely uptake of EID decreased if babies’ mothers were not on ART before pregnancy, and their ART center was far away from the PCR facility. Among the samples sent for EID from respective ART centers, 33% of the results did not reach the mother [11].

This long TAT and high proportion of nonreceipt of result defeated the actual purpose of EID. Identification and addressing the risk factors for long TAT will help the program in ensuring that the results are conveyed in a timely manner to the mother. Therefore, this study was conducted to determine the factors associated with long TAT or nonreceipt of results by mothers among HIV-exposed babies under the IHC program (2013–2015) whose samples were collected for EID (PCR) within 9 months of age.

## Methods

### Study design

This was a cohort study involving record review of routinely collected data. EID cascade included the step from sample collection for PCR to receipt of PCR results by mother.

### Setting

#### General setting

Myanmar is situated in South-East Asia and has 15 states and regions. They are administratively divided into districts and further into 412 townships/subtownships. It has an estimated population of 51 million, of whom 70% live in rural areas [12].

#### EID under IHC program

The Union implements the IHC program through a public–private–patient partnership model and provides HIV care based on the national guidelines [13]. It is involved in 15 ART centers across ten cities in five states and regions. Among them, twelve ART centers have a pediatrician and provide EID. Data of HIV-exposed babies is maintained as a separate database in all these centers except in Sagaing and Kalaw.

Blood samples for EID (dry blood spot for DNA PCR or frozen plasma in ice box for RNA PCR) and PCR results are transported between ART center and PCR facility using public transport. This process is supported by The Union logistics team. PCR is routinely run within 2 weeks of sample receipt at PCR facility, subject to receipt of a sufficient number of samples. The results are given to mother/care giver during the scheduled follow-up visit at the ART center (one month after sample collection). Further details of EID under the IHC program have been reported elsewhere [11].

### Patient population

Ten ART centers out of 12 ART centers (Sagaing and Kalaw were excluded) providing EID under the IHC program, Myanmar, were included in the study. Among all HIV-exposed babies enrolled under the IHC program in the ten ART centers between January 2013 and December 2015, babies whose samples were collected for PCR test before the age of 9 months were the patient population.

### Data variables, sources of data, and data collection

Records of HIV-exposed babies were extracted from the electronic databases at ART centers and PCR facility, and merged using a unique identifier. They were tracked from sample collection to result receipt by the mother.

In addition to key socio-demographic and clinical characteristics (Tables 1 and 2), data variables extracted included IHC code (unique identifier); dates of birth, enrollment, sample collection and result receipt by mother; and distance of ART center from baby’s township and PCR facility. The source of data for sample collection date was the database at PCR facility. We assumed that the date of result receipt by the mother was her follow-up date immediately after the result was dispatched. Distance was calculated using google maps (www.google.com./maps).

**Table 1.** Socio-demographic characteristics of HIV-exposed babies (age <9 months at enrollment) whose samples were sent for PCR test under the Integrated HIV Care program, Myanmar, 2013–2015.

Characteristics		n	%
Total		1000	100
Sex of baby			
	Male	516	52
	Female	482	48
	Not recorded	2	<1
Age of mother at enrollment			
in years	<18	3	<1
	18–24	162	16
	25–34	471	47
	≥35	134	13
	Not recorded	230	23
Mother’s marital status			
	Single mother	8	1
	Married	627	63
	Widow	38	4
	Divorced/separated	10	1
	Not recorded	317	32
Mother’s education status			
	Literate	606	61
	Not literate	145	15
	Not recorded	249	25
Mother’s employment status			
	Employed	268	27
	Unemployed/homemaker	397	40
	Not recorded	335	34

HIV: human immunodeficiency virus; PCR: polymerase chain reaction test used for early infant diagnosis of HIV.

**Table 2.** Clinical and programmatic characteristics of HIV-exposed babies (age <9 months at enrollment) whose samples were sent for PCR test under the Integrated HIV Care program, Myanmar, 2013–2015.

Characteristics		N	%
Total		1000	100
Mother’s baseline CD4			
cells/mm^3^	<100	82	8
	100–349	351	35
	≥350	343	34
	Not recorded	224	22
Mother’s predelivery			
treatment status	On ART before pregnancy	485	49
	On PMTCT	248	25
	Not on ART and PMTCT	267	26
Mode of delivery			
	NSVD	339	34
	AVD	2	<1
	LSCS	625	63
	Not recorded	34	3
Place of delivery			
	Hospital	851	85
	Private	3	<1
	Home	115	11
	Not recorded	36	4
**Programmatic factors**		**N**	**Median (IQR)**
Distance of ART center from patient’s township in kilometers		1000	10 (3, 46)
Distance of PCR facility from patient’s ART center in kilometers		1000	105 (3, 162)

HIV: human immunodeficiency virus; PCR: polymerase chain reaction test used for early infant diagnosis of HIV; ART: antiretroviral therapy; PMTCT: prevention of mother to child transmission; NSVD: normal spontaneous vaginal delivery; AVD: assisted vaginal delivery; LSCS: lower section caesarian section; IQR: Interquartile range.

### Analysis and statistics

Data extracted in MS EXCEL were imported into EpiData analysis software (version 2.2.2.183, EpiData Association, Odense, Denmark) for descriptive and unadjusted analysis. Predictive multivariable modeling was done using STATA (version 12.1 STATA Corp., College Station, TX, USA).

Frequency and proportion (categorical variables) and median with interquartile range (IQR) (distance) were used to summarize patient population characteristics. Age and CD4 count (continuous variables) were categorized as described in Tables 1 and 2.

TAT was classified based on the median value of 7 weeks. Outcome of interest was the composite of delayed (≥7 weeks) TAT or nonreceipt of result by mother. Unadjusted analyses were performed to assess the factors associated with delayed TAT or nonreceipt of result by mother when compared with TAT <7 weeks. For modeling, distance was categorized based on the median value. Variables with a p-value of <0.2 in the unadjusted analysis, after excluding variables with multicollinearity (mother employment and literacy status), which was assessed using variance inflation factor, were fitted in Poisson regression with robust variance estimates (enter method). HIV-exposed babies with missing variables were excluded from the regression analysis: a complete case analysis was done. Unadjusted and adjusted relative risks (RR and aRR) were reported with 95% CI.

## Results

**Figure 1.**
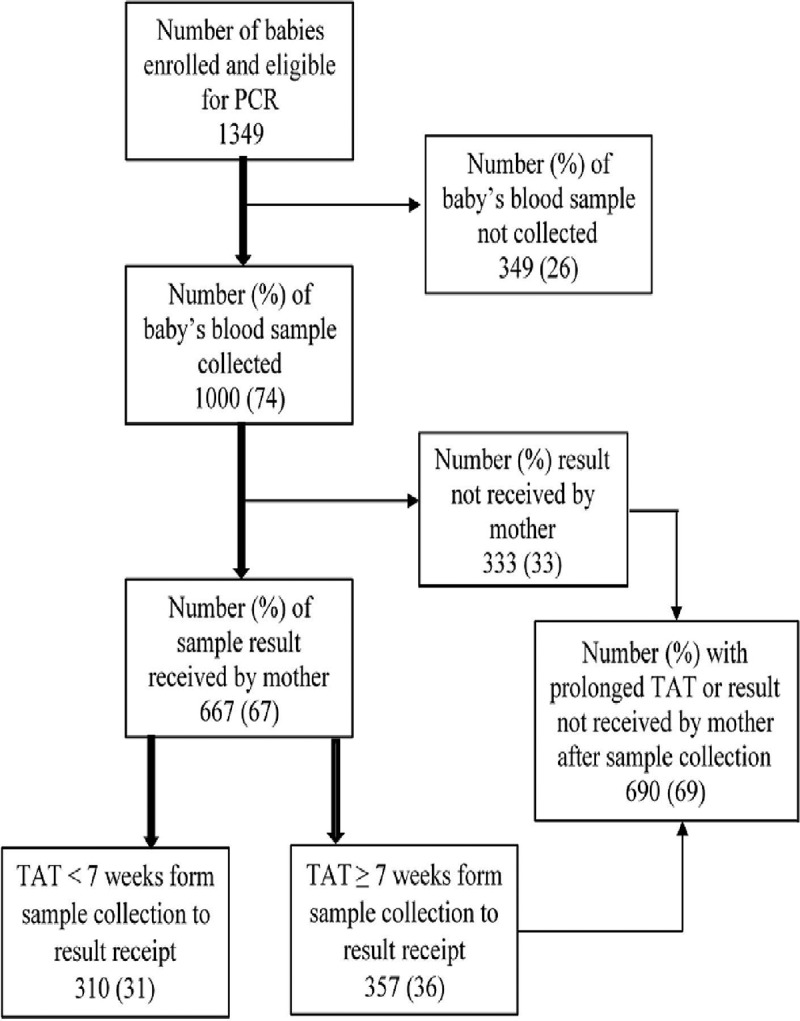
Flow chart for early infant diagnosis cascade among HIV-exposed babies whose samples were sent for a PCR test (at age <9 months) under the integrated HIV care program, Myanmar, 2013–2015. HIV: human immunodeficiency virus; PCR: polymerase chain reaction test used for early infant diagnosis of HIV; ART: antiretroviral therapy.

Among the 1461 babies enrolled into IHC during the study period, 1349 babies were eligible for PCR at <9 months of age, and blood samples of 1000 babies were sent for PCR, for inclusion in the analysis (Figure 1). Their socio-demographic, clinical, and programmatic characteristics have been summarized in Tables 1 and 2.

Of the 1000 babies, TAT was <7 weeks and ≥7 weeks in 310 (31%) and 357 (36%) babies respectively; the results were not received by the mother in 333 (33%) cases. Long TAT (≥7 weeks) or nonreceipt of results was seen in 69% (690/1000) babies (Figure 1).

The risk of long TAT or nonreceipt of results was >50% across all subgroups and was not significantly different among those with a positive (77%) and negative (67%) PCR result (p = 0.15). In an unadjusted analysis, factors associated with long TAT or nonreceipt of results were: female sex of baby, mother being nonliterate, mother’s CD4 count of 100–350 cells/mm^3^, mother being neither on ART before pregnancy nor on PMTCT during pregnancy, and long distance (≥105 km) between ART center and PCR facility (Table 3).

**Table 3.** Factors associated with prolonged TAT (>7 weeks) or result not received (outcome) by mothers of HIV-exposed babies whose samples were sent for PCR test (at age <9 months) under integrated HIV care program, Myanmar, 2013–2015.

Variable	Type	Evaluated ^ (n)	Outcome (%)	RR (95%CI)	aRR (95%CI)**
Total		1000	680 (68)	–	–
**Socio-demographic**					
Sex of baby	Female	482	342 (71)	1.1 (1.0, 1.2)*	1.1 (0.9, 1.2)
	Male	516	336 (65)	Ref.	Ref.
Age of mother in	<18	3	0 (0)	–	–
Years	18–24	162	102 (63)	1.1 (0.9, 1.3)	1.1 (0.9, 1.4)
	25–34	471	283 (60)	1.1 (0.9, 1.3)	1.1 (0.8, 1.3)
	≥35	134	76 (57)	Ref.	Ref.
Mother’s marital	Single mother	8	6 (75)	1.4 (0.9, 2.0)	–
Status	Married	627	349 (56)	Ref.	–
	Widow	38	20 (53)	1.0 (0.7, 1.3)	–
	Divorced/separated	10	7 (70)	1.3 (0.8, 1.9)	–
Mother’s education	Literate	606	334 (55)	Ref.	–
status	Not literate	145	114 (79)	1.4 (1.3, 1.6)*	–
Mother employed	Yes	268	137 (51)	Ref.	–
	No/homemaker	397	231 (58)	1.1 (1.0, 1.3)	–
**Clinical factors**					
Mother’s baseline	<100	82	52 (63)	1.0 (0.8, 1.2)	1.0 (0.8, 1.2)
CD4 cells/mm^3^	100–349	351	192 (55)	0.9 (0.8, 0.97)*	0.8 (0.7, 0.9)*
	≥350	343	218 (64)	Ref.	Ref.
Mother’s pre-	On ART	485	305 (63)	Ref.	Ref.
delivery treatment	On PMTCT	248	166 (67)	1.1 (1.0, 1.2)	0.9 (0.7, 1.0)
status	None	267	209 (78)	1.2 (1.1, 1.4)*	1.0 (0.8, 1.2)
Mode of delivery	NSVD	339	228 (67)	Ref	–
	AVD	2	2 (100)	–	–
	LSCS	625	422 (68)	1.0 (0.9, 1.1)	Ref.
Place of delivery	Hospital	851	577 (68)	Ref.	–
	Private	3	3 (100)	–	–
	Home	110	72 (66)	1.0 (0.8, 1.1)	–
**Programmatic factors**					
Distance from ART	≥10 km	502	342 (68)	1.0 (0.9, 1.1)	–
center to pt. residence	<10 km	498	338 (68)	Ref.	
Distance from ART	≥105 km	491	397 (81)	1.5 (1.3, 1.6)*	1.2 (1.1, 1.4)*
center to laboratory	<105 km	509	282 (56)	Ref.	Ref.

^Missing records not included in analysis; *statistically significant, p < 0.05; ** Adjusted RR using modified poisson regression (enter method); factors with unadjusted p < 0.2 were included in model; mother employment/literacy status were excluded due to high collinearity.

HIV: human immunodeficiency virus; PCR: polymerase chain reaction test used for early infant diagnosis of HIV; EID: early infant diagnosis; ART: antiretroviral therapy; PMTCT: prevention of mother to child transmission; NSVD: normal spontaneous vaginal delivery; AVD: assisted vaginal delivery; LSCS: lower section caesarian section; pt.: patient; km: kilometer; RR: relative risk.

In an adjusted analysis (232 HIV-exposed babies were excluded from adjusted analysis due to missing variables), babies with a mother’s CD4 count of 100–349 cells/mm^3^ had a 20 percent lower risk of long TAT or nonreceipt of results when compared with babies with a mother’s CD4 count of ≥350 cells/mm^3^ [aRR (0.95 CI): 0.8 (0.7, 0.9)]. A long distance between the ART center and PCR facility (≥105 km) had a 20% higher risk of long TAT or nonreceipt of results when compared with distance <105 km [aRR (0.95 CI): 1.2 (1.1, 1.4)] (Table 3).

## Discussion

Under the IHC program in Myanmar, more than two-thirds of HIV-exposed babies whose sample was sent for PCR received their results after seven weeks of sample collection or did not receive the results at all. Among these, only two results were not dispatched from the PCR facility [11]. Prolonged TAT or nonreceipt of results was uniformly high over all subgroups. It was significantly lower among those with a mother’s CD4 count of 100–349 cells/mm^3^ at enrollment and significantly higher among babies with their ART centers far away from PCR facility.

### Strengths and limitations

This was the first study from Myanmar to systematically study factors associated with long TAT within EID on a large scale. All the HIV-exposed babies whose samples were sent for PCR (at <9 months of age) within IHC were included in this study. The study was operational in nature, utilizing existing resources without additional funding. Hence, the findings are representative and present a true picture of the program. We used Google maps for calculating the distance [14], as this was not routinely recorded in the program.

The limitations were similar to our previous study [11]. Dates for sample receipt and testing at PHL were not routinely recorded. The date of result dispatch at PHL was available but could not be used because if a baby was tested more than once, the date of dispatch for the most recent result was recorded due to overriding of an earlier dispatched date by the database (34 instances out of 1 000 samples). We assumed that the immediate date of the follow-up visit in records after result dispatches was the date of result receipt by the mother. However, it might have been possible that the mother followed up on the designated date but the results were not available. Hence, we could not look at the cascade of TAT in depth. We could not assess the independent effect of the mother’s literacy and employment status on long TAT or nonreceipt of result by mother due to a high collinearity. Due to the secondary nature of the data, information on variables was missing in some records (Tables 1 and 2). This was also partly because many mothers were not enrolled into IHC.

### Key findings

Though long TAT or nonreceipt of result was uniformly high among subgroups, we identified two important risk factors.

Mothers with a CD4 count of 100–349 cells/mm^3^ had higher chances of being on ART than mothers with a CD4 count ≥350 cells/mm^3^. During 2013–2014, a mother who was enrolled in IHC with a CD4 count <350 cells/mm^3^ was eligible for ART. Mothers on ART before pregnancy (CD4 count of 100–349 cells/mm^3^ at enrollment) must have been on a regular follow-up and aware of the need for returning to receive test results for her baby when compared with other mothers living with HIV. However, mothers with counts lower than 100 (having a higher chance of being on ART, even more than mothers with counts 100–349 cells/mm^3^) had similar risks when compared with mothers with a CD4 count ≥350 cells/mm^3^, and this was intriguing. It is possible that there were additional factors, not captured routinely within IHC, that could have led to this.

Long distance between ART center and PCR facility was the second risk factor. This was obvious because public transportation was used for paper-based communication of results. Mobile phones were not used systematically to convey the results before paper-based results reached the ART center. Surprisingly, distance between ART center and patient’s township had no effect on the risk of long TAT or nonreceipt of result.

Various other studies have described TAT across different countries, which ranged from three weeks to 13 weeks, but few had systematically analyzed factors associated with long TAT or nonreceipt of results [8,15–19]. In Uganda, it was found that longer turnaround times, clinic entry point, and age at sample collection may be associated with receipt of infant HIV test results [8].

#### Recommendations for policy and practice

Universal ‘test and treat’ (as per WHO recommendation) for people living with HIV needs to be implemented in Myanmar [20]. This will reduce transmission to baby, reduce TAT, and improve timely receipt of result by the mother as the mother is on ART. Other recommendations to reduce TAT like point of care technology providing PCR testing, systematic implementation of innovative ideas to communicate EID results (a short messaging service has been started in 2016 by PCR facility), and use of courier services for transporting samples should be introduced [15,21]. The recommendations of our previous studies are similar to those of the current study. Therefore, this study further lends support to the recommendations of our previous study [11].

It has been shown in rural Tanzania that simple interventions designed by a pediatric HIV nurse, applied sequentially, resulted in reduction of TAT [16]. Therefore, further operational research should be explored in future to test the effectiveness of interventions to reduce TAT. Qualitative research is recommended to explore mothers’ and program personnel perspectives.

Routine data collection within IHC program needs to improve, considering large number of records with missing data, and it is urgently needed to integrate with national data base for routine data collection (Tables 1 and 2). The program should systematically record the dates of receipt of sample and testing at the PCR facility. The date of result dispatch should be recorded for each sample.

## Conclusion

This operational research identified that once the sample is sent for EID, there was a high proportion with long TAT or nonreceipt of results by mothers. Point-of-care testing for EID may reduce TAT/nonreceipt of results by mothers. Health system, laboratory, and logistic factors such as sample transportation, laboratory procedures, and result dispatching associated with long TAT should be further explored.

## Paper context

This is a follow-up paper of a recently published article titled ‘Early infant diagnosis of HIV in Myanmar: Call for innovative interventions to improve uptake and reduce turnaround time’. In an earlier study, we reported a low uptake of EID and a long median TAT from sample collection for PCR test to result receipt by babies’ mother. This study assesses the risk factors of long TAT and/or nonreceipt of results by mothers.
